# Overcoming language barriers in healthcare: A protocol for investigating safe and effective communication when patients or clinicians use a second language

**DOI:** 10.1186/s12913-015-1024-8

**Published:** 2015-09-10

**Authors:** Renata F. I. Meuter, Cindy Gallois, Norman S. Segalowitz, Andrew G. Ryder, Julia Hocking

**Affiliations:** School of Psychology and Counselling, Queensland University of Technology, Brisbane, QLD Australia; School of Psychology, The University of Queensland, St Lucia, QLD Australia; Department of Psychology, Concordia University, Montreal, QC Canada; Culture and Mental Health Research Unit, Jewish General Hospital, Montreal, QC Canada

## Abstract

**Background:**

Miscommunication in the healthcare sector can be life-threatening. The rising number of migrant patients and foreign-trained staff means that communication errors between a healthcare practitioner and patient when one or both are speaking a second language are increasingly likely. However, there is limited research that addresses this issue systematically. This protocol outlines a hospital-based study examining interactions between healthcare practitioners and their patients who either share or do not share a first language. Of particular interest are the nature and efficacy of communication in language-discordant conversations, and the degree to which risk is communicated. Our aim is to understand language barriers and miscommunication that may occur in healthcare settings between patients and healthcare practitioners, especially where at least one of the speakers is using a second (weaker) language.

**Methods/Design:**

Eighty individual interactions between patients and practitioners who speak either English or Chinese (Mandarin or Cantonese) as their first language will be video recorded in a range of in- and out-patient departments at three hospitals in the Metro South area of Brisbane, Australia. All participants will complete a language background questionnaire. Patients will also complete a short survey rating the effectiveness of the interaction. Recordings will be transcribed and submitted to both quantitative and qualitative analyses to determine elements of the language used that might be particularly problematic and the extent to which language concordance and discordance impacts on the quality of the patient-practitioner consultation.

**Discussion:**

Understanding the role that language plays in creating barriers to healthcare is critical for healthcare systems that are experiencing an increasing range of culturally and linguistically diverse populations both amongst patients and practitioners. The data resulting from this study will inform policy and practical solutions for communication training, provide an agenda for future research, and extend theory in health communication.

## Background

Barriers to effective and equitable healthcare can result from linguistic differences between patients and clinicians [1–3]. Increasingly, healthcare professionals include migrants whose first language (L1) is not the majority language [4]. Patients who are linguistic minority migrants, a group also increasing in number, must similarly use a second language (L2) during their healthcare encounters, or rely on the availability and accuracy of an interpreter. Thus growing numbers of patients using a country’s healthcare system do not share an L1 with their practitioner and vice versa. Language discrepancies may result in increased psychological stress and medically significant communication errors for already anxious patients, something to which patients in language-congruent encounters (i.e. shared L1) are less vulnerable [5]. Moreover, it is not just language that can cause barriers to equitable healthcare: inequities inherent in the social dynamic of the patient-practitioner encounter are well documented, and these inequities occur independent of whether L1 is shared [6]. Understanding language in the context of a medical encounter is thus critical for understanding the problems that might result when patients and healthcare practitioners speak a different L1. This research is designed to explore these potential barriers in a natural healthcare communication setting, across a range of hospital in- and out-patient departments.

When communicating the details of a diagnosis or treatment, it is crucial to convey accurately the likelihood of the associated risk factors [7, 8]. Failure to communicate properly the seriousness of risk can have negative consequences: patients may fail to comply with instructions or elect not to have potentially life-saving treatment. Although there has been much information published on communication of risk between patients and healthcare practitioners in healthcare situations, this research has focused predominantly on language-congruent situations. It is not clear how health-related risk is appropriately and accurately conveyed to a patient when their first language is discordant with that of the practitioner and the wider community. There is evidence that miscommunication is more likely to occur when clinicians use an inadequately mastered L2 and cannot correctly convey certain nuances of risk and certainty [9]. Complicating matters further, people from different cultural groups describe pain and distress quite differently: culturally-specific terms, expressions, or metaphors can be difficult to navigate even when language competence is high [10]. Also, when clinicians lack the linguistic and cultural skills needed and interpreters are not available [11], patients may have to rely on medically inexperienced, bilingual relatives or non-medical staff, compromising quality of care and worsening health outcomes for migrant communities.

### Theoretical framework

There are at least three theoretical approaches to understanding why communication problems arise in language-discrepant medical communication settings. One is a psycholinguistic approach discussed by Segalowitz and Kehayia [12], in which the focus is on the way in which speakers direct the other person’s focus of attention to key elements of their message by using semantic and syntactic features of the language to package the message appropriately, and on the listener’s ability to infer the speaker’s intention. A second theoretical approach considers the conversational dynamics of patient-doctor interactions [13]. The focus here is the power relation differences between doctor and patient, and how language use both reflects these relationships and serves as a tool for manipulating them. Little is known regarding the social dynamics that operate in language-discrepant healthcare contexts. Here we will apply a third theoretical approach, namely the framework of Communication Accommodation Theory (CAT) [14, 15]. This approach has a particular relevance for comparing language-discrepant and language-congruent communication. In theoretical terms, CAT proposes that 1) speakers attempt to converge (or not) their manner of speaking, to accomplish important social goals around attaining social approval, identity etc.; 2) the extent to which speakers converge reflects in part the need for communication efficiency; 3) convergence is viewed as positive and normative; and 4) divergence in manner of speaking reflects a specific intention to do so, and is normally perceived negatively. CAT thus provides a useful framework for examining the dynamics of patient-practitioner communication, especially when at least one of the speakers uses an L2. In such cases, an inability to achieve convergence (i.e. to appear more similar in speech) can affect how the speakers perceive not only each other, but also the quality of the working relationship between them e.g. [16–18]. The relevant research goal here is to identify what specific impact language discrepancy has on accommodation, and what the consequences are for patient-practitioner communication.

In summary, our study will produce a corpus of authentic patient-practitioner communication that will be explored systematically, using a combination of qualitative and quantitative analyses. This will allow us to establish the linguistic elements of the interactions that may contribute to a language barrier, as well as communication-based factors that hinder or facilitate language discordant conversations. Because Chinese (Mandarin and Cantonese) speakers comprise the largest group who speak language other than English in the home in the Greater Brisbane region (2.4 %: http://www.censusdata.abs.gov.au), we focus on conversations between English and Chinese native speakers, in an English-speaking hospital system. Video recordings of actual patient-practitioner interactions will be acquired and analysed using both qualitative and quantitative methods, including computer-based automatic analyses of communication flow. This multi-methods approach will allow us to explore the dynamics of patient-practitioner communication, including the use of critical linguistic expressions related to the communication of risk, in the context of concordant and discrepant language interactions. We predict that: 1) language-discrepant situations will be characterised by communicative interactions that are both qualitatively and quantitatively poorer than language-congruent situations; and 2) these differences will be greater the lower the proficiency in the L2, and the more crucial the need for accurate communication of risk.

## Methods/Design

### Data collection

The research will take place at three hospitals located in the Metro South area of Brisbane, Australia: The Logan, Princess Alexandra and Queen Elizabeth II Hospitals. These hospitals were selected for their large size and comprehensive range of departments, and based on the demographic composition of their patient populations. Naturalistic conversations that occur between a patient and health practitioner, during an on-site hospital appointment, will be video recorded. These conversations will be obtained from a range of hospital in- and out-patient departments, in order to obtain a selection of health-related problems with different levels of patient risk. Departments considered appropriate for data collection include facilities for video recording in a quiet, private environment, as well as the ability to identify potential patient participants in advance (i.e. not those departments seeing or admitting emergency patients). The optimum length of appointment to obtain data appropriate for transcribing and analysing was determined as 15–20 min. Broad consultation with Directors of Nursing at each site, and with Nurse Unit Managers (NUMs), determined that the optimal process of initial patient and practitioner identification will be via the NUMs. An equivalent number of interactions will be recorded between participants who share the same L1, and those who do not share the same L1, in order to assess the effect of linguistic ability on the quality of the interaction and the communication of health-related information.

### Participants

Patients and practitioners who speak either English or Chinese (Mandarin or Cantonese) as their L1 will be invited to participate, with initial recruitment facilitated by the NUMs. Chinese was chosen as the other language because it is the most common language spoken in South East Queensland after English. Depending on patient demographics, practitioners will be recorded in at least two interactions with patients. Patients will participate in one recorded interaction only. The patient cohort will comprise a minimum of 40 monolingual English and 40 bilingual (or polyglot) Chinese-English speakers. These patients will form dyads with 40 practitioners (20 monolingual English, 20 bilingual or polyglot Chinese-English), such that each practitioner will be recorded with at least two patients, one with whom they share an L1 (i.e. English L1 patient - English L1 practitioner or Mandarin L1 patient - Mandarin L1 practitioner) and one for whom the L1 is incongruent (i.e. English L1 practitioner - Mandarin L1 patient or English L1 patient - Mandarin L1 practitioner). We anticipate that most conversations will be in English. However, it is likely that practitioner and patient may resort to their common L1 (if not English) when that facilitates their communication. Conversations assisted by an interpreter will also be recorded. The practitioners will be recruited from multiple professions, including clinical nurses, midwives and pharmacists, thus allowing evaluation of a range of conversational dynamics.

### Materials and procedure

Practitioners willing to participate in the research will be administered an information sheet and consent form, as well as a Language Background Questionnaire (LBQ), in advance of the video recording. Patients who are either language concordant or discrepant will be identified in a number of ways. First, NUMs will search the Queensland Health “Hibiscus” system (HBCIS: Hospital Based Corporate Information System) to identify patients with upcoming appointments who have identified themselves as Chinese, or requiring a Chinese interpreter, thus enabling the researchers to introduce themselves to the patient when they arrive for their appointment to seek their consent. Second, practitioners who have consented to take part will identify potential patients and contact the research team directly to inform them of a potential participant. Third, poster advertising will be used across the hospital and in local press to inform the public about the research and request that they get in touch should they be visiting the hospital as a patient and are interested in taking part in the study.

All information, consent and questionnaire forms will be available in a choice of English, Traditional Chinese or Simplified Chinese, allowing patients to select their preferred language. After providing informed consent, patients will participate in a video recording session during their hospital appointment. It should be noted that audio-only recording will be used if video recording is not possible. Basic language and L2 proficiency background information will be obtained using the LBQ (including self-rated proficiency for L1 and L2), for both patients and practitioners. This questionnaire was based on the work of two of the authors (RM and NS), described in [19] and adapted from [20]. In addition, patients will be asked to complete a short questionnaire to rate the perceived effectiveness of communication with the practitioner after their appointment has ended.

### Exclusion and inclusion criteria

Patients and practitioners who speak more than two languages (e.g. Cantonese, Mandarin, and English) will be included in the study, as will those individuals (patients and practitioners) who identify English as their dominant language but who acquired Cantonese/Mandarin in childhood and speak it at home. Patients who are identified as having requested the assistance of an interpreter will be recorded, provided that they have consented to take part in the study. There will be no video recording of appointments where patients are expected to receive a physical examination by the practitioner.

### Data analysis

All conversations will be transcribed in preparation for both qualitative and quantitative analyses. The transcripts will be analysed using Discursis [21], which will look at periods of engagement between patient and practitioner, with a focus on the extent to which communicative needs were met. In terms of communication content, Discursis will also allow us to investigate the extent to which expressions of likelihood and risk are used, and the extent to which they are linked to changes in convergence. Paralinguistic features such as tempo or pitch will also be available for analysis, for example convergence in register i.e. to what extent do patient and practitioner pitch their communication at the same level.

Data from the LBQ (e.g. L2 proficiency, language of training), as well as the practitioner/patient’s conversation, will be used to inform the metrics derived from the Discursis analysis. The metrics derived from these conversations can then be used to compare with the LBQ and the outcome measures from the post-appointment questionnaire for both qualitative and quantitative analyses. The qualitative analysis will look at accommodation, as well as target the occurrence of specific adverb phrases and how they operate in the context of discussing health risk.

### Ethics

Ethical approval for the study has been granted from the Queensland University of Technology and University of Queensland Human Research Ethics Committees, and the Metro South Hospital Research Ethics Committee of the Queensland Government. Site specific approvals have been obtained for the Princess Alexandra, Queen Elizabeth II and Logan Hospitals.

## Discussion

The study will yield, for the first time, information about the flow of language-concordant and language-discrepant communication, as a function of whether the clinician or the patient is the L2 speaker. Understanding L2 communication in a health context is important because inadequate communication may have negative consequences, including increased psychological stress to the patient, medically significant communication errors and misunderstandings of potential health risk. Understanding the linguistic and cultural elements of these interactions will help us understand how potentially serious outcomes can arise during language-discrepant interactions, and address these at both theoretical and practical levels.

A small pilot study determined that a brief questionnaire regarding the quality of the interaction captured the patient’s experience better if they were allowed to rate the quality of the conversation with their practitioner. Using these ratings, we will be able to look at the degree to which the patient’s perceptions of the interaction match the conversational agreement revealed using Discursis. Any discrepancy between subjective perceptions and actual conversational agreement may then be linked to particular linguistic features, the patient’s and/or practitioner’s L2 status, or the level of risk communicated (with a higher risk level possibly requiring more complex language or terms).

One of the practical challenges of this study is that of obtaining sufficient numbers of each possible practitioner-patient combination. Because L2 speakers are allowed, and indeed encouraged, to seek assistance from an interpreter, there are likely to be a number of conversations where an interpreter may be present and involved to a greater or lesser degree in supporting the communication between practitioner and patient. These conversations will be analysed separately to enable an analysis of the impact of interpreter presence. Another challenge is posed by the need to balance language concordant and discordant conversations with the need to capture conversations that vary in the extent to which their content focuses on risks to the patient. As discussed above, a wide number of departments in each hospital are targeted to mitigate the problem of obtaining conversations that do not vary sufficiently in patient-risk content. The bilingual speakers, be they practitioners or patients, are likely to have mastered more than the two languages focused on here. Knowledge and use of more than two languages also may impact on the speaker’s ability to accommodate, their linguistic sophistication, and their cultural awareness (which may impact on the conversational dynamics). The background information obtained will allow further analysis of the possible differential impact of bilingual versus multilingual language use on the clarity of conversations.

In summary, to address the problem of language barriers successfully, we must know when they are most likely to arise and what their specific nature is. To do so, new research methods must be developed, and a theoretical framework formulated to generate research questions and guide research. This study will allow us to:

1. explore new ways to systematically study — at a micro-level of analysis — the nature of language barriers in healthcare communication;
2. address specific aspects of language barriers in healthcare communication in a way that will inform the design of language training programs for clinicians; and
3. articulate a research agenda for future theoretical, empirical, and applied work aimed at overcoming language barriers in healthcare delivery (e.g. in indigenous communities; rural/remote healthcare).

## Abbreviations

CAT: Communication Accommodation Theory
L1: First, or native, language
L2: A second, non-primary, language
LBQ: Language Background Questionnaire
NUMS: Nurse Unit Managers

## References

[1] Chu C (1998). Cross-cultural health issues in contemporary Australia. Ethnicity Health.

[2] Jacobs E, Chen AHM, Karliner LS, Agger-Gupta N, Mutha S (2006). The need for more research on language barriers in health care: A proposed research agenda. Milbank Q.

[3] Murray SB, Skull SA (2005). Hurdles to health: immigrant and refugee health care in Australia. Aust Health Rev.

[4] Hawthorne L (2001). The globalisation of the nursing workforce: barriers confronting overseas qualified nurses in Australia. Nurs Inq.

[5] Bowen S (2000). Language barriers in access to health care.

[6] Wodak R, Brown K (2006). Medical discourse: doctor-patient communication. Encyclopaedia of language and linguistics.

[7] Schenker Y, Wang F, Selig SJ, Ng R, Fernandez A (2007). The impact of language barriers on documentation of informed consent at a hospital with on-site interpreter services. J Gen Intern Med.

[8] Gillotti C, Thompson T, McNeilis K (2002). Communicative competence in the delivery of bad news. Soc Sci Med.

[9] Roberts GW (1994). Nurse/patient communication within a bilingual health care setting. Br J Nurs.

[10] Ryder AG, Ban LM, Chentsova-Dutton YE (2011). Towards a Cultural–Clinical Psychology. Soc Personal Psychol Compass.

[11] Gany F, Yogendran L, Massie D, Ramirez J, Lee T, Winkel G, Diamond L (2013). Leng J: “Doctor, what do I have?” Knowledge of cancer diagnosis among immigrant/migrant minorities. J Cancer Educ.

[12] Segalowitz N, Kehayia E (2011). Exploring the determinants of language barriers in health care (LBHC): Toward a research agenda for the language sciences. Can Modern Lang Review.

[13] Candlin CN, Candlin S (2003). Health care communication: A problematic site for applied linguistics research. Ann Rev Appl Linguist.

[14] Gallois C, Giles H, Joens E, Cargile AC, Ota H, Wiseman RL (1995). Accommodating intercultural encounters: Elaborations and extensions. Intercultural Communication Theory.

[15] Gallois C, Ogay T, Giles H, Gudykunst WB (2005). Communication accommodation theory: A look back and a look ahead. Theorizing about intercultural communication.

[16] Gasiorek J, van de Poel K (2012). Divergent Perspectives on Language-Discordant Mobile Medical Professionals’ Communication with Colleagues: An Exploratory Study. J Appl Commun Res.

[17] Segalowitz N (1976). Communicative incompetence and the non-fluent bilingual. Can J Behav Sci Revue canadienne des sciences du comportement.

[18] Segalowitz N (2010). Cognitive bases of second language fluency.

[19] Meuter RFI, Allport A (1999). Bilingual Language Switching in Naming: Asymmetrical Costs of Language Selection. J Mem Lang.

[20] Freed BF, Dewey DP, Segalowitz N, Halter R (2004). The language contact profile. Stud Second Lang Acquis.

[21] Angus D, Smith A, Wiles J (2012). Conceptual recurrence Plots: Revealing patterns in human discourse. IEEE Trans Vis Comput Graph.

